# Tracing metabolic flux to assess optimal dietary protein and amino acid consumption

**DOI:** 10.1038/s12276-022-00817-w

**Published:** 2022-09-08

**Authors:** Robert R. Wolfe, Il-Young Kim, Sanghee Park, Arny Ferrando

**Affiliations:** 1 Department of Geriatrics, Center for Translational Research in Aging & Longevity, Donald W. Reynolds Institute on Aging, University of Arkansas for Medical Sciences, Little Rock, AR USA; 2 Department of Molecular Medicine, College of Medicine, Gachon University, Incheon, Korea; 3 Korea Mouse Metabolic Phenotyping Center, Lee Gil Ya Cancer and Diabetes Institute, Gachon University, Incheon, 21999 Korea

**Keywords:** Translational research, Metabolic diseases

## Abstract

There is a general consensus that a dietary protein intake of 0.8 g protein/kg/day will prevent symptoms of protein deficiency in young, healthy individuals. However, individuals in many physiological circumstances may benefit from higher rates of dietary protein intake. Stable isotope tracer methodology enables a variety of approaches to assessing the optimal dietary protein intake in humans. In this paper, we present an overview of a variety of tracer methods, with a discussion of necessary assumptions, as well as the clinical circumstances in which different methods may be preferable. Although we discuss the nontracer method of nitrogen balance, which has historically been used to estimate dietary protein requirements, this paper primarily focuses on tracer methods for estimating dietary protein and essential amino acid requirements under different physiological conditions. We will explain the following approaches: isotopic measurement of urea production; the arterial-venous tracer balance method; measurement of the fractional synthetic and breakdown rates of muscle protein; the indicator and the direct amino acid oxidation methods; and different approaches to measuring whole-body protein synthesis and breakdown. The advantages and limitations of each method are discussed in the context of the optimal approaches for use under different circumstances.

## Nutrition: Optimising monitoring of dietary protein intake

Both clinical and practical considerations should inform how dietary protein intake is tracked in individuals. Little is known about optimal dietary protein intake in humans under different physiological conditions including disease states, prompting a review of current monitoring methods by Robert Wolfe at the University of Arkansas, Little Rock, USA, and co-workers. Monitoring nitrogen balance provides estimates of dietary protein and essential amino acid (EAA) levels, but requires several days of strict diet control. Various techniques monitor the uptake or fate of a specific tracer molecule in the body, but some are very invasive. The less invasive single EAA infusion methods provide rapid results and are valuable for monitoring immediate response to a meal, or in studies comparing protein intakes to a person’s baseline over time. Using two methods concurrently could improve accuracy of results.

## Introduction

There are thousands of proteins in the body. All of these body proteins are in a state of turnover, meaning that they are constantly being metabolically degraded (protein breakdown) and resynthesized. The rate of resynthesis of body proteins must match the rate of protein breakdown over the course of the day to maintain physiological homeostasis. For this to occur, there must be sufficient amino acid precursor availability to match the amount of amino acid release in the process of protein breakdown. However, a fraction of the amino acids released in the process of protein breakdown are oxidized, making them unavailable as precursors for protein synthesis. Some of the amino acids required for the replacement of oxidized amino acids can be produced by metabolic processes in the body (nonessential amino acids, NEAAs), while others (essential amino acids, EAAs) can only be replaced by dietary intake.

EAAs are conventionally derived from dietary protein. The question of how much dietary protein is sufficient to supply the EAAs necessary to enable protein synthesis to balance the rate of protein breakdown has been a focus of nutrition research for more than 100 years. While the minimal amount of dietary protein required to maintain protein balance in young, healthy adults is generally well agreed upon (0.8 g protein/kg body wt/day), several circumstances, such as aging, severe illness or trauma, exercise training, etc., may change the required amount of dietary protein intake. Furthermore, research suggests that there may be a distinction between the minimal protein requirement and the optimal rate of protein intake (e.g., ref. ^1^). Determining the optimal rate of dietary protein and EAA consumption in a variety of circumstances has proven to be challenging. The physiological end point of maintaining a balance between protein synthesis and breakdown has traditionally been the lean body mass as a reflection of muscle mass.

However, it may take weeks for changes in lean body mass to be reliably measured. Because of the lengthy periods of time often required for nutritional interventions to be reflected in physiological and body composition changes, surrogate approaches using stable isotope tracers

have been used to estimate the optimal rate of dietary protein intake. In this review, we will discuss a variety of approaches that may be used to assess dietary protein and essential amino acid requirements in different physiological circumstances. We will discuss the advantages and limitations of tracer methods in the context of specific applications for which each method is best suited.

## Nitrogen balance

Although the nitrogen balance (NB) technique is not a tracer method, it is useful to consider in the context of determining optimal rates of dietary protein consumption, since NB has been considered the gold standard for close to 100 years and is the basis for current nutritional guidelines^2^. The theory underlying the NB technique proposes that the amount of N consumption required to balance the amount of N lost from the body represents the amount of dietary N intake needed to maintain lean body mass. The NB method has been utilized to estimate protein requirements in normal, healthy individuals, and there is an enormous database in that demographic. However, there are important limitations to the NB technique that make it less valuable in determining optimal rates of dietary protein intake in populations other than young adults in good health. The difficulty of accurately measuring both N intake and all sources of N loss had resulted in a great deal of variability in NB data. In addition, the NB analysis conventionally uses urinary N excretion to represent N loss from the body, but N can be lost by other routes, including feces, sweat and wound exudates. Even if the technical issues are overcome, several days of precise dietary control are needed, during which time the experimental conditions must be constant. Most importantly, changes in NB do not always correspond to changes in lean body mass over time^3^.

## Urea production

Stable isotope-labeled urea and the traditional primed-constant infusion technique can be used to measure urea production, and this value can be used to estimate the net rate of protein breakdown^4^. Protein breakdown and amino acid oxidation result in nitrogen being delivered to the liver in the form of alanine and glutamine at rates in excess of their proportionate contents in body protein, as well as in excess of the requirements for those amino acids as precursors for hepatic protein synthesis. Excess N is incorporated into urea and excreted from the body in urine. Approximately 85–90% of nitrogen excretion is in the form of urea^5^, and consequently, the rate of urea production and excretion is a good reflection of the net rate of protein breakdown.

Isotopic studies of urea metabolism date to the 1950s^6,7^. In these early studies, higher rates of urea production were obtained when the carbon or oxygen in urea was labeled with a stable isotope tracer than when an N-labeled urea tracer was used. The discrepancy in rates of urea production with different tracers was due to the underestimation of total urea production resulting from the use of ^15^N_2_-urea as a tracer and the measurement of total nitrogen enrichment. When ^15^N_2_-urea is infused, singly labeled urea is produced from recycled ^15^N^8^. Thus, when the enrichment of singly and doubly labeled urea is distinguished, the total rate of urea production can be calculated from the doubly labeled (infused) enrichment, while the singly labeled urea can be used to calculate the amount of recycling of urea N. The rate of total urea production minus the recycling of urea nitrogen is a direct reflection of whole-body protein breakdown in the postabsorptive state^4^. Whole-body protein breakdown can also be calculated using the ^15^N_2_-urea method in the fed state if N intake in the meal is considered.

The use of isotopically labeled urea to estimate the net rate of protein breakdown is limited by the fact that not all N loss from the body occurs in the form of urea. Consequently, the rate of net protein breakdown will be underestimated by the labeled urea tracer method. On the other hand, there are significant advantages to the tracer method for measuring urea production. Most importantly, reliable values can be obtained within 4 h of primed-constant tracer infusion. Furthermore, acute increases (in response to alanine infusion) and decreases (in response to glucose infusion) in urea production can be determined over 1 h or less once an isotopic steady state has been achieved^9^. Thus, although the precise value of net protein breakdown may be underestimated by the tracer method, comparative studies quantifying the response to acute perturbations, such as varying levels of dietary protein in a meal, can be reliably performed.

## The arterial-venous difference approach

The measurement of the arterial-venous (A-V) balance of an EAA across a muscle bed reflects net muscle protein synthesis. Net muscle protein synthesis is an important component of the total lean body mass, and the response of net muscle protein to differing levels of dietary protein or amino acid intake has been used as an end point in a variety of studies aiming to determine the optimal rate of dietary intake. The general principle of the A-V balance technique is that the net uptake or release of an essential amino acid that cannot be either produced or oxidized in muscle (e.g., phenylalanine) is a direct reflection of the net balance between synthesis and breakdown. The A-V balance of phenylalanine can be measured across the leg or the forearm. In each case, arterial samples are needed. The site of arterial sampling to measure the A-V difference is not important since all of the arterial blood will have the same amino acid concentration. In contrast, venous sampling must be collected from the region of interest. In the case of the leg muscle, the femoral vein is catheterized, whereas a deep forearm vein is catheterized for measurement of the A-V balance across the forearm. The A-V difference of the amino acid (e.g., phenylalanine) is multiplied by the measured rate of blood flow to the tissue to determine the net balance. Rates of muscle protein synthesis and breakdown can be calculated when performed in conjunction with a constant infusion of isotopically labeled phenylalanine^10^. When labeled phenylalanine is infused, samples of the intracellular fluid are obtained (by needle biopsy) in addition to arterial and venous samples, and then a variety of additional parameters can be calculated, including inward and outward transport rates. Additionally, the extent to which protein synthesis is derived from amino acids recycled from protein breakdown as opposed to amino acids taken up from plasma can be distinguished^10^. Phenylalanine kinetics can be extrapolated to protein kinetics by accounting for the proportional contribution of phenylalanine to muscle protein (approximately 4%). The specific tracer species of phenylalanine is not important, since the principle of the method relies on dilution of the infused tracer rather than the metabolic fate of the labeled phenylalanine^10^.

**Fig. 1:**
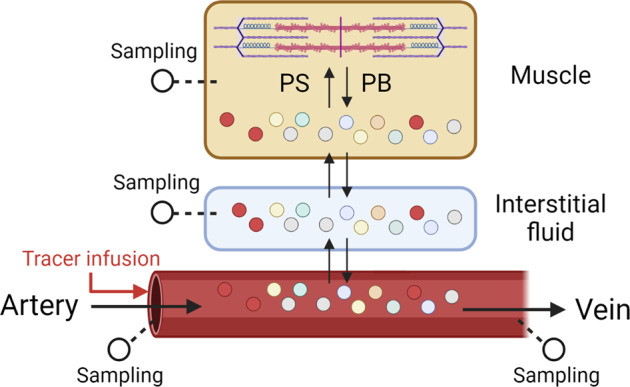
Schematic representation of the A-V balance method. Red circles represent tracers. This approach is useful for measuring leg muscle protein synthesis and breakdown as well as amino acid transmembrane transport. The same model can be used across the forearm, except that the inability to obtain a muscle biopsy will limit the accuracy of the estimation of protein synthesis and breakdown, and transmembrane transport cannot be quantified. PS protein synthesis; PB protein breakdown. This figure was created with BioRender.com.

The A-V model using labeled tracers has been validated both theoretically and empirically^10–12^. The A-V tracer method was performed on the first day of a period of 28 days of complete bed rest. Activity and nutritional intake were absolutely controlled throughout, thereby minimizing the impact of confounding variables usually encountered when extrapolating results from acute metabolic studies of muscle metabolism to long-term changes in lean body mass. Bed rest induced a loss of leg lean mass calculated by the A-V model that was not significantly different from the actual loss determined by DEXA measurement^12^. Thus, the A-V model provides an accurate measurement of muscle protein metabolism. The kinetic data are physiologically relevant, and the A-V approach has the advantage that a steady state is not required^13^. It is therefore possible to continuously determine muscle protein kinetics over an entire 24-h period^14^ while quantifying acute responses (such as those occurring after a meal) within the context of the 24-h experiment. A schematic representation of the A-V model is shown in Fig. 1.

While there are many advantages of the A-V model, there are practical limitations. In both the leg and forearm models, it is assumed that muscle accounts for the entire observed response, although skin and bone are also drained by the same veins that drain the muscle. This complication is relatively minor with the leg model and can be addressed quantitatively by additionally measuring the intracellular phenylalanine concentration and enrichment in a skin sample obtained by biopsy^10^. In contrast, the inability to distinguish skin from muscle metabolism is of more concern with the forearm model, as the ratio of skin to muscle is much greater in the forearm than in the leg. Furthermore, the forearm model is limited by the inability to obtain a muscle biopsy. Importantly, regarding use in human subjects, the A-V model is invasive, particularly across the leg. Arterial and femoral vein catheterizations are needed, and ideally, muscle biopsies are obtained as well. These procedures can be performed without adverse events^15^ but nonetheless present a significant limitation to the widespread application of the method. From a technical standpoint, the results are highly dependent on the accurate measurement of blood flow rate, which can be problematic. Thus, while the A-V model can reliably predict changes in muscle mass over time, there are technical problems that limit its widespread use.

## Muscle fractional synthetic and breakdown rates

Simultaneous measurement of the muscle fractional synthetic rate (FSR) and fractional breakdown rate (FBR) enables quantification of the net gain of muscle protein in response to different levels of protein or amino acid intake. The muscle FSR is determined by the rate of incorporation of a tracer amino acid into muscle protein over time. Any amino acid precursor can be used to determine the FSR, but since the FBR can only be determined by analyzing an EAA that cannot be oxidized in muscle, phenylalanine is typically used as the tracer if the FSR and FBR are to be determined simultaneously. The tracer amino acid is conventionally infused or injected as a bolus, but the amino acid precursor can also be labeled endogenously from ingested deuterated water.

Muscle tissue is typically collected by needle biopsy, but the recently described “virtual biopsy” technique enables calculation of the muscle FSR from the increase in enrichment of creatine kinase M-type isolated from plasma (CK-M), since plasma CK-M arises almost entirely from muscle tissue^16^. FSR calculation requires that the tracer incorporation per unit time be corrected by precursor enrichment to account for the amount of tracer given and its dilution by the unlabeled (trace) endogenous counterpart. The true precursor enrichment is generally reflected by enrichment of the free amino acid tracer in the intracellular fluid of the muscle sample in which the bound amino acid enrichment is determined, since this reflects the true precursor for synthesis^17^. Alternatively, if deuterated water is used to enrich a precursor amino acid, the true precursor enrichment can be estimated from the enrichment of body water, which can be determined on samples of plasma, saliva, or urine^16^. FSR determination is the most commonly used tracer method for determining the response to varying levels of protein or amino acid intake.

The point at which increasing protein intake causes no further increase in FSR has been interpreted as reflective of protein requirements. However, use of the FSR alone is an inadequate approach because it ignores the role of changes in protein breakdown with increasing amounts of protein intake, as NB is the balance between protein synthesis and breakdown. For this reason, simultaneous measurement of the FSR and FBR provides the best assessment of the net gain in muscle protein in response to a meal.

**Fig. 2:**
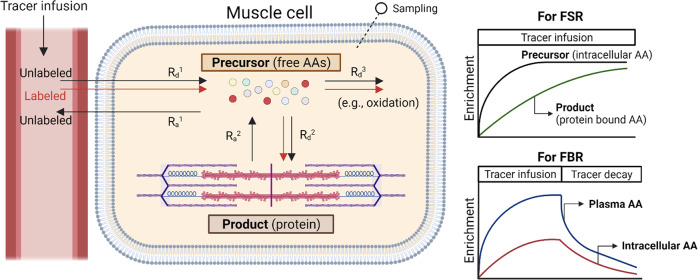
Schematic representation of the measurement of protein FSRs and FBRs. Tracer amino acids can be administered either as a primed-constant infusion or as a bolus. Alternatively, the precursor amino acid can be labeled from orally ingested deuterated water. R_a_ rate of appearance of the tracee; R_d_ rate of disappearance of the tracee; FSR fractional synthesis rate; FBR fractional breakdown rate; AAs amino acids. This figure was created with BioRender.com.

The determination of FBR is based on the concept that the decay in enrichment in the intracellular fluid of muscle after a bolus injection or cessation of a constant infusion of an amino acid tracer is a direct reflection of the rate of appearance of unlabeled tracee amino acids from protein breakdown, provided that the tracer amino acid is neither produced nor oxidized in muscle tissue. Phenylalanine is typically used to measure FBR since it satisfies these criteria. Interpretation of the decay in enrichment of the intracellular pool of tracer is complicated by the fact that unlabeled phenylalanine can also enter the intracellular space from plasma. The appropriate calculation enables distinction of the appearance of unlabeled phenylalanine from protein breakdown and influx from plasma^18^, thereby enabling calculation of the FBR. A schematic representation of the measurement of protein FSR and FBR is shown in Fig. 2.

Advantages of evaluating optimal protein and amino acid consumption rates by measuring the FSR and FBR include the following: both parameters are determined on the exact same muscle sample; there is a large database of results from FSR measurements; and the FSR/FBR approach is easier than the A-V model from a technical standpoint because while biopsies are required for both models, arterial and deep-venous samples are not required for the FSR/FBR model. However, the nature of the data generated from determination of FSR/FBR, however, has some limitations when applying results to the estimation of protein requirements. When estimating the extent of conversion of ingested protein to lean body mass, it is preferable to deal with absolute rates. However, FSR and FBR are expressed as %/unit of time. These units mean that the calculated values reflect the percentage of the total muscle protein pool in the body that is synthesized and broken down per unit time. Conversion to actual amounts of protein synthesis and breakdown requires multiplication by the total muscle protein mass (i.e., pool size) represented by the single site muscle biopsy. When this conversion is performed by combining the FSR/FBR measurements with an estimation of the total muscle protein mass, the results are similar to those obtained by the A-V balance technique^19^. Since the A-V model and the FSR/FBR model rely on entirely different assumptions, the similarity of the resultant data tends to confirm the validity of each approach. However, in contrast to the FSR, which can be measured over a prolonged time frame (i.e., 24 h or longer) to determine the total daily response to the amount of protein intake, the FBR can only be measured over a period of 30–90 min because of the speed of dilution of the intracellular enrichment of the tracer. The restriction on the FBR measurement period limits the practical utility of the method in terms of determining the total daily requirement or optimal intake of protein.

## Indicator amino acid oxidation (IAAO) and direct amino acid oxidation (DAAO) methods

The IAAO method has been used to estimate both protein and amino acid requirements in different circumstances, primarily in normal subjects^20^. The concept of this approach is based on the fact that the fate of the tested indispensable (essential) amino acid (usually phenylalanine for determination of protein requirements) is either oxidation or protein synthesis. Therefore, determination of the rate of phenylalanine oxidation using a ^13^C-labeled tracer indirectly estimates the extent of incorporation of phenylalanine into protein (i.e., protein synthesis). Consumption of progressively greater amounts of dietary protein (in the case of estimating protein requirements) stimulates protein synthesis correspondingly (as reflected by decreases in phenylalanine oxidation) until a plateau is reached. The breakpoint defining the maximal rate of protein synthesis in response to increasing doses of dietary protein corresponds to a plateau in the excretion of ^13^CO_2_, and that breakpoint theoretically defines protein requirement^20^. When determining the dietary requirement for an individual EAA, the subject ingests a surfeit of free amino acids in the profile of egg protein minus the test EAA. Then, progressively increasing amounts of the test EAA are added to the amino acid mixture, with a corresponding decrease in phenylalanine oxidation (reflecting an increase in protein synthesis), until a breakpoint is reached. As in the case of determining the total protein requirement with the IAAO method, the breakpoint in ^13^CO_2_ defines the dietary requirement for the test EAA.

The IAAO method has practical advantages, as it requires only peripheral tracer infusion and collection of peripheral blood samples and breath samples. Additionally, determination of the response at different levels of protein or EAA intake requires only a few hours. The primary technical limitation is the need to determine the rate of total CO_2_ production by indirect calorimetry, which can be quite variable compared to the measurement of ^13^CO_2_ enrichment. The IAAO method involves testing the response to mixtures of free amino acids, which are rapidly absorbed completely, whereas the protein in actual dietary food sources may be incompletely absorbed and result in lower peak concentrations of EAAs than when free amino acids are consumed. In addition, the IAAO method ignores the possible role of changes in protein breakdown in response to increasing levels of protein or EAA intake. As described above, the net gain or loss of muscle reflects the balance between protein synthesis and breakdown. Once the maximal stimulation of protein synthesis is achieved, further increases in protein intake cause progressive reductions in protein breakdown^21^. The resulting response of net protein balance with a progressive decrease in breakdown while synthesis is constant is the same as when synthesis is stimulated in the absence of a change in breakdown. Thus, the net protein balance continues to increase in a linear manner at levels of protein intake well above those that maximize synthesis^21^.

Since the inhibition of protein breakdown becomes a significant issue primarily at relatively high rates of protein intake^22^, the IAAO method probably defines a reasonable value for the estimated average (basal) requirement for dietary protein. However, the method may be less accurate in defining requirements under circumstances in which an amount of protein intake beyond the basal requirement might be desirable in certain circumstances.

The practical application of the IAAO method in “field” conditions may be enhanced by using the urinary enrichment of the tracee precursor (e.g., phenylalanine) as opposed to the blood enrichment to quantify flux^23^. The protocol must be modified if urinary enrichment is used to calculate flux to achieve an equilibrium in enrichment, which will take longer than in blood. Although using urinary enrichment rather than blood enrichment of the tracee precursor makes the IAAO method even less invasive, it is likely that the responsiveness to differences in protein intake is diminished. Thus, phenylamine flux does not differ over a wide range of dietary protein intake when calculated using urinary phenylalanine enrichment^23^, which is an unlikely result.

The DAAO method is based on the same principle as the IAAO method, but rather than using oxidation of an indicator amino acid to estimate the effect of the test meal on protein synthesis, the test amino acid itself is the tracer^24^. For example, the dietary requirement for leucine would involve the infusion of 1-^13^C-leucine and the collection of ^13^CO_2_ at graded levels of leucine intake in the context of the intake of a surfeit of the other EAAs. In this example, leucine oxidation will not increase if intake is below the requirement level, and when the requirement level is reached, the rate of ^13^CO_2_ production will increase linearly with progressively greater amounts of leucine intake.

While the underlying assumptions of the IAAO and DAAO methods are basically the same, the DAAO method has the technical challenge of needing a different tracer for each EAA tested. As in the case of the IAAO, the possible impact of suppression of protein breakdown as part of the anabolic response to the progressively increasing intake of dietary protein or EAA is not taken into account, and the response to mixtures of free amino acids may overestimate the response to food sources of dietary protein.

## Whole body protein synthesis and breakdown

Whole-body protein turnover refers to the pooled rates of protein synthesis and breakdown of all body proteins. Whole-body protein kinetics have played an important role in assessing optimal levels of dietary protein intake. Whereas whole-body protein models have been criticized because they do not allow the quantitation of individual pathways, it is reasonable to assess nutritional requirements at the whole-body level because the process of food consumption and utilization involves the whole body. Whole body protein kinetics are particularly useful for quantification of the response to a single meal. Different whole-body turnover methods of quantifying the response to consumption of dietary protein have been analyzed in detail (e.g., ref. ^25^). All methods of determining the whole-body protein kinetic response to nutrient intake require assumptions that can potentially affect the validity of the calculations. As a result, the optimal approach depends on the experimental paradigm.

### General principles of whole-body protein models

**Fig. 3:**
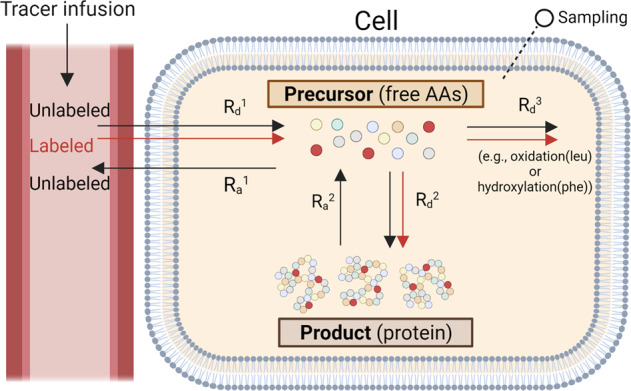
Schematic representation of the general principle of the measurement of whole-body protein kinetics. R_a_ rate of appearance of the tracee into the blood; R_d_ rate of disappearance of the tracee from the blood. This figure was created with BioRender.com.

Tracers may label the entire nitrogen (N) pool (e.g., ^15^N-glycine, ^15^N-alanine) or trace the kinetics of an individual essential amino acid (EAA) that cannot be produced in the body.

(e.g., 1-^13^C-leucine, ^2^H_5_-phenylalanine). The general principles of the assessment of whole-body protein kinetics using stable isotope tracers are schematically presented in Fig. 3. The EAA tracer enables quantification of the rate of appearance of the tracee in the blood, which in the fasted state is considered to be a direct function of whole-body protein breakdown^19^.

Subtraction of the rate of irreversible loss (e.g., oxidation) of the tracee from its rate of appearance derives a value which is assumed to be a direct function of whole-body protein synthesis. Net whole-body protein balance is equal to protein synthesis minus breakdown^19^. The challenge when assessing the response to dietary protein intake with a whole-body protein turnover model is how to distinguish the contribution of absorbed tracee from that released by the breakdown of body proteins.

### N-flux model of protein kinetics

An ^15^N-labeled amino acid tracer is used to label the entire N pool of the body.

Historically, ^15^N-glycine was most commonly used to label the whole-body N pool. Theoretically, either urinary urea or ammonia enrichment can be used to calculate nitrogen flux, although urea has generally been the end-product of choice because it is the predominant form in which N is excreted. The residual label in urea at the end of the study period (i.e., enrichment x concentration), multiplied by the volume of distribution of urea (total body water, TBW), must also be accounted for in the calculation of N-flux. TBW can be directly measured, either by stable isotope dilution or by bioimpedance analysis. Alternatively, for healthy young adults, TBW can be assumed to be 0.764 x lean body mass if the lean body mass is known^26^ or 0.6 x body weight if the lean body mass is not known^27^. The impact on the calculated rates of whole-body protein synthesis and breakdown of using an assumed value for total body water is generally small.

There is also residual ^15^N in ammonia in TBW, but the amount of ^15^N in ammonia is generally insufficient to significantly affect the calculated rates of protein kinetics.

Determination of whole-body protein kinetics by the N-flux model requires the assumption that the entire N pool is equally labeled with ^15^N from the tracer via transamination reactions. Unfortunately, ^15^N is not evenly distributed throughout the entire N pool when ^15^N-glycine is used as a tracer. If ^15^N was equally distributed, the enrichments of the end products of N flux (urea and ammonia) would be equal, but ^15^N-urea enrichment is generally approximately 50% higher than ammonia enrichment in the postabsorptive state. This difference has led to the speculation that glycine is preferentially converted to urea as opposed to other amino acids^28,29^. Nonetheless, there is strong empirical support for the concept of a single-body N pool that is reflected by the enrichment of the urea pool. Stein et al.^30^ compared the rates of protein synthesis calculated by analyzing the enrichment of the nitrogen end-products to the incorporation of ^15^N-glycine into the entire homogenized carcass of rats. Their data indicated good agreement between the indirect method of measuring whole-body N flux using urea enrichment as the end-product and the direct (tissue incorporation) method, thereby providing strong empirical support for the assumption of a single N pool that is reflected by urea enrichment. We have observed that the discrepancy between urea and ammonia enrichment is minimized with the use of ^15^N-alanine as a tracer^31^. Alanine plays an important role in the interorgan transfer of amino nitrogen from the muscle, gut, and kidney for hepatic urea production, is extensively involved in transamination reactions^32,33^ and therefore is the preferable tracer for the N-flux model.

Quantification of the anabolic response to dietary protein consumption by the N-flux method requires accounting for the contribution of the exogenous tracee to the total measured rate of appearance of the tracee. Thus, protein breakdown in the postprandial state is calculated as the total N-flux minus the amount of dietary N consumed. While all N consumed may not be absorbed, the amount of error from this assumption is unlikely to be large. Furthermore, the N-flux approach to the determination of the response of endogenous protein kinetics to dietary protein consumption has the great advantage that no assumption is required regarding splanchnic clearance of ingested N; the N-flux value includes all splanchnic protein metabolism.

The most limiting feature of whole-body N-flux determination is that rapid changes in protein kinetics, such as those occurring after a meal, cannot be determined. Isotopic equilibration throughout the body nitrogen pool requires too much time for the method to be useful in acute studies. For this reason, models of protein kinetics based on the use of a single essential amino acid tracer have become popular.

### Single EAA models of whole-body protein kinetics

The principal advantage of EAA models, compared to the N-flux model, is that with appropriate priming of the relevant pools, it is possible to collect reliable rates of protein synthesis and breakdown within 90 min of the start of tracer infusion. Because of the short time required and rapid tracer responses, evaluation of the acute response to various perturbations, including meal feeding, is possible. A further advantage of the EAA models is that data can be presented as the change from baseline, which is particularly useful when the baseline values of individual subjects in a study differ, which is often the case in clinical conditions such as injury or serious illness.

A variety of EAA tracers with different end products have been used with this basic model. The most popular choices have been 1-^13^C-leucine as an infused tracer and ^13^CO_2_ as the oxidation product and ^2^H_5_-phenylalanine as an infused tracer and hydroxylation of phenylalanine to tyrosine as the product of the irreversible loss of phenylalanine. The leucine model has the advantage of being able to approximate precursor enrichment for oxidation by measuring the plasma enrichment of alpha-ketoisocaproic acid (KIC) due to the intracellular equilibrium between KIC and leucine^34^. The leucine model has the technical disadvantage of necessitating the collection of breath samples to measure both the rate of total CO_2_ excretion as well as the expired CO_2_ isotopic enrichment. The phenylalanine model has the advantage of not requiring any breath samples but presents the disadvantage that there is no surrogate analogous to KIC that reflects the intracellular enrichment of phenylalanine in the liver (where hydroxylation occurs). Both acute and longer-term responses can be quantified at different levels of protein intake with the single EAA models. Although it is always desirable to perform tracer studies in a physiological steady state, this condition is not mandatory for the single amino acid tracer method. Thus, the approach is well suited to the determination of optimal protein intake in difficult circumstances, such as hospitalization in the intensive care unit. Additionally, acute responses to meal feeding, as well as the response to exercise or other physiological perturbations, can be quantified over time. The single-EAA methodology is very responsive to varying levels of protein intake. In addition, specific protein FSRs can be determined simultaneously by isolating the protein of interest from the blood or by obtaining tissue biopsies.

**Fig. 4:**
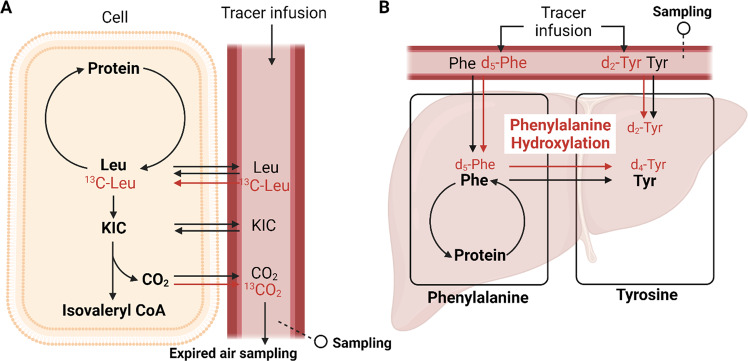
Schematic representation of the single EAA model. The examples are using either 1-^13^C-leucine (**A**) or ^2^H_5_-phenylalanine (**B**) as tracers. Leu leucine; KIC alpha-ketoisocaproate; Phe phenylalanine; Tyr tyrosine. This figure was created with BioRender.com.

We have previously discussed the various assumptions underlying the EAA models (e.g., refs. ^19,25^). It is assumed that the kinetics of a single EAA are assumed to be representative of all EAAs and that the measured flux of the EAA being traced reflects its proportional contribution to the composition of whole-body protein. The validity of these assumptions, as well as other assumptions implicit in the EAA model, is supported by the empirical evidence that in the postabsorptive state, the calculated rates of whole-body protein synthesis and breakdown are similar with different EAA tracers and end products. This includes the infusion of ^15^N-lysine, with ^15^N-urea as the end-product^35^; the infusion of 1-^13^C-lysine, with ^13^CO_2_ as the end-product^31^; the infusion ^of^ 1-^13^C-leucine, with ^13^CO_2_ as the end-product^36^; and the infusion ^of 2^H_5_-phenylalanine, with hydroxylation to tyrosine as the end-product^37^. 1-^13^C-Leucine and ^2^H_5_-phenylalanine have most commonly been used. The use of these tracers is illustrated in Fig. 4. The controversial issue with the single EAA model is how to best quantify protein kinetics following consumption of a meal containing dietary protein.

### Quantifying protein kinetics in the fed state with the EAA model

Consideration must be given to the contribution of absorbed amino acids from dietary protein to the total flux rate of the tracee to calculate whole-body protein breakdown using the EAA model. The rate of appearance of exogenous tracee (exogenous Ra) must be subtracted from the total R_a_ as measured in the peripheral plasma to derive the rate of endogenous protein breakdown. The calculated rate of whole-body protein synthesis, on the other hand, does not require the endogenous and exogenous Ra’s to be distinguished, and therefore, an error in determining the exogenous R_a_ will affect only the accuracy of the calculated rate of protein breakdown. Consequently, an error in the determination of exogenous R_a_ will translate to an error not only in the value of protein breakdown but also in the net balance.

There are two basic approaches for quantifying exogenous R_a_ in the context of the EAA model: the intrinsically labeled protein method and the bioavailability approach^25,38^.

### Intrinsically labeled protein approach for quantifying exogenous R_a_

The intrinsically labeled protein approach involves producing a dietary protein containing the same tracer amino acid as is infused but labeled differently, thereby enabling differentiation of the ingested source of the labeled amino acid from the infused tracer. The rate of appearance of exogenous tracers in peripheral blood is divided by the isotopic enrichment of the intrinsically labeled protein to calculate the rate of appearance of exogenous tracees in peripheral blood.

That rate is subtracted from the total R_a_ of the tracee to calculate the endogenous R_a_ and thus the rate of protein breakdown. The validity of this approach hinges on the assumption that the appearance of the ingested tracer amino acid in the peripheral blood quantitatively reflects the appearance of its unlabeled counterpart. In other words, the enrichment of the labeled amino acid in the intrinsically labeled protein must not be diluted from the point of ingestion to the point of sampling in the peripheral blood by any process other than protein breakdown. However, the isotopic enrichment of ingested tracer in an intrinsically labeled protein is diluted in the gastrointestinal tract (GIT) by the digestive process, as well as by recycling of tracer^25^. Complications caused by loss of label prior to reaching the peripheral blood can be minimized by creating a steady state in the enrichment of peripheral blood by sip feeding of the exogenous protein, but loss of tracer in the gastrointestinal tract in the process of digestion and absorption cannot be avoided. As a result, the intrinsically labeled protein approach underestimates the amount of absorbed tracee that appears in peripheral blood and thus overestimates the contribution of protein breakdown to the total Ra, as measured by the intravenous infusion of tracer. Since the calculation of protein synthesis does not involve the R_a_ of exogenous tracee, the net balance between protein synthesis and breakdown will be underestimated by the intrinsically labeled protein approach.

### Bioavailability method for estimating exogenous R_a_

The exogenous rate of appearance following consumption of dietary protein using the bioavailability approach is estimated from the amount of protein ingested, the true ileal digestibility (TID) of the dietary protein, and the irreversible loss of the tracee in the splanchnic bed. The approach can be used with any EAA model of protein kinetics, but the phenylalanine method is advantageous because the fraction of absorbed tracee cleared by the splanchnic bed can be directly measured as the fraction of phenylalanine flux hydroxylated to tyrosine in the liver^25^. One advantage of the bioavailability approach is that the response to a combination of a variety of proteins can be quantified. In addition, a physiological steady state is not needed, meaning that the method is well suited for quantifying the response to a meal. Furthermore, the complication of the intrinsically labeled protein approach caused by the secretion and digestion of endogenous protein is avoided because this process does not cause any change in net tracee absorption. However, only the total anabolic response can be determined because only the total contribution of exogenous phenylalanine to the peripheral circulation can be estimated, not the rate at which it is absorbed.

**Fig. 5:**
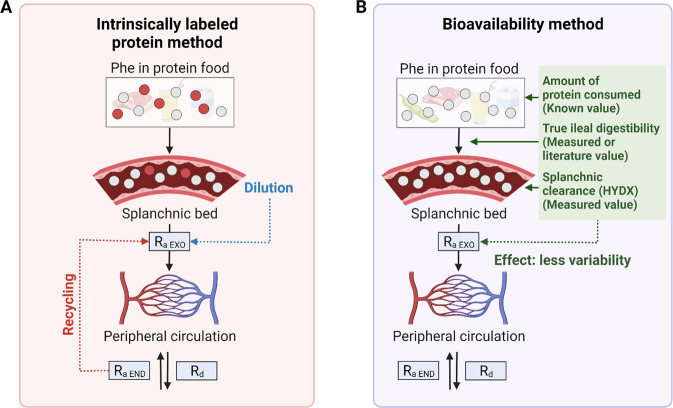
Quantification of exogenous R_a_ in the context of the EAA model. **A** The intrinsically labeled protein method introduces the tracer into the body as a component of the ingested protein. The method is complicated by potential isotopic exchange in the process of digestion and during transit through the splanchnic bed. **B** The bioavailability method accounts for exogenous R_a_ by correcting the total protein intake for the true ileal digestibility of the tracee EAA. It is assumed that there is no gain or loss of intestinal protein in the process of digestion and absorption. Phe phenylalanine; red circle labeled Phe; gray circle unlabeled Phe; R_a_ rate of appearance of the tracee; R_d_ rate of disappearance of the tracee; R_a_ EXO, R_a_ of exogenous Phe; R_a_ END, R_a_ of endogenous Phe; HYDX hydroxylation. This figure was created with BioRender.com.

There are three significant assumptions underlying the estimation of the total appearance of ingested phenylalanine in the peripheral circulation according to the bioavailability model. First, the measurement of the percentage of phenylalanine uptake hydroxylated to tyrosine in the liver is assumed to accurately reflect the splanchnic clearance of absorbed phenylalanine prior to appearance in the peripheral circulation. Second, it is assumed that the rate of synthesis of gastrointestinal tract proteins balances the rate of digestion of endogenous proteins secreted into the GIT. Third, the true ileal digestibility of the tracee in the protein meal must either be assumed or measured independently. The first assumption is reasonable since the irreversible loss of phenylalanine occurs only in the liver^39^. There are no data addressing the second assumption, and it is possible that there is a net gain of gastrointestinal protein following a protein meal. If true, the result would be an underestimation of total whole-body protein synthesis. The third assumption could potentially introduce error into the measurement of the amount of the contribution of absorbed phenylalanine to the appearance of phenylalanine in the peripheral circulation, depending on the accuracy of the assumed value of true ileal digestibility. Any error introduced by this assumption would be random and would not systematically bias the calculated rates of whole-body protein synthesis and breakdown. The theory underlying the intrinsically labeled protein approach and the bioavailability approach are shown in Fig. 5.

## Summary and conclusions

The optimal amount of dietary protein under any circumstance would best be determined by varying the level of protein consumption and controlling all other aspects of nutrient intake and physiological condition for a sufficient time to quantify changes in a relevant end point, such as lean body mass or physical function. However, end points influenced by dietary protein consumption usually change slowly over time, making it very difficult to minimize the influence of confounding variables. There are a variety of methods that provide surrogate measures of physiological end points, and each method has advantages and disadvantages. Nitrogen balance is a reasonable approach in very controlled situations, and there is an abundant database. Unfortunately, this method generally requires several days of dietary control, and the results are often quite variable. As a result of the limitations of the traditional nitrogen balance technique, tracer methods have been developed that can be applied to determine the optimal rates of protein and amino acid consumption.

The determination of urea production using stable isotope tracer methodology enables calculation of net protein balance within a few hours but provides no information regarding protein synthetic and breakdown rates. The A-V balance technique provides quantification of net protein balance in muscle and, when combined with stable isotope tracer methodology, enables the quantification of muscle protein synthesis, muscle protein breakdown and amino-acid transmembrane transport rates. However, the method is limited by its invasiveness and by the difficulty of quantifying leg blood flow. Additionally, it is difficult to extrapolate from muscle metabolism to whole body protein requirements. Determination of the muscle FSR and FBR is the only approach based entirely on the direct sampling of muscle tissue, but (as with the A-V model), FSR/FBR results are difficult to extrapolate to protein requirements at the whole-body level. The IAAO method is noninvasive and easily applied to measure the response to differing levels of intake of dietary protein as well as individual essential amino acids but is limited by being only a reflection of protein synthesis; consequently, the IAAO method may underestimate the net protein balance at higher rates of dietary protein intake when suppression of protein breakdown may further enhance the net protein balance. The measurement of whole-body protein synthesis and breakdown rates enables quantification of both the acute response to meal feeding as well as other, integrated responses.

Whole-body protein synthesis and breakdown approaches encompass the entire physiological response to dietary protein intake and, in the case of the single EAA models, enable quantification of the acute response to meal ingestion. It is also possible to combine methods, as multiple stable isotope tracers can be given simultaneously. For example, it is possible to determine muscle protein FSR/FBR contemporaneously with application of the single EAA infusion approach.

None of these tracer methods are unequivocally the best for determining the optimal rates of protein intake in all populations and circumstances. The results from studies using the nitrogen balance approach have been the most widely used by advisory committees developing nutrition guidelines, but this method is difficult to perform accurately, requires control of experimental conditions that are often impractical, and has no direct interpretation. Tracer methods have advantages over the nitrogen balance method, particularly under dynamic physiological circumstances, such as exercise or critical illness. The preferable tracer method depends not only on the physiological circumstances but also on practical aspects, such as the time frame of interest, the degree of tolerable invasiveness, and even practical issues, such as the cost of tracers and the type of analytical equipment available. For example, the ^15^N-flux and IAAO methods are suitable for studies in children, in whom only limited invasiveness can be tolerated. The tracer determination of urea production and the A-V model can be useful in clinical settings, including the intensive care unit, where the necessary catheters may already be in place, and the variability in the baseline condition of the subjects makes measurement of the acute change from baseline a preferable approach. The FSR/FBR methodology is particularly relevant for studies focused on physical function and exercise performance. The single-EAA infusion models are well-suited for determining the response to a meal. Regardless of the physiological state and the tracer method selected, it is advisable to consider the validity and reasonable ranges of the necessary assumptions and calculate the upper and lower bounds of protein kinetics. It is also advantageous to use two methods concurrently that have different assumptions. Finally, when feasible, the results from acute tracer studies should be confirmed with a corresponding outcome trial.
